# Interhemispheric Inhibition during Mental Actions of Different Complexity

**DOI:** 10.1371/journal.pone.0056973

**Published:** 2013-02-25

**Authors:** Nicolas Gueugneau, Marco Bove, Laura Avanzino, Agnès Jacquin, Thierry Pozzo, Charalambos Papaxanthis

**Affiliations:** 1 Université de Bourgogne, Unité de Formation et de Recherche en Sciences et Techniques des Activités Physiques et Sportives, Dijon, France; 2 Institut National de la Santé et de la Recherche Médicale (INSERM), Unité 1093, Cognition, Action et Plasticité sensorimotrice, Dijon, France; 3 Department of Experimental Medicine, Section of Human Physiology and Centro Polifunzionale di Scienze Motorie, University of Genoa, Genoa, Italy; 4 Italian Institute of Technology, Genoa, Italy; 5 Institut Universitaire de France (IUF), Paris, France; 6 Service de Neurologie, Faculté de Médecine de Dijon, Dijon, France; University of Ottawa, Canada

## Abstract

Several investigations suggest that actual and mental actions trigger similar neural substrates. Yet, neurophysiological evidences on the nature of interhemispheric interactions during mental movements are still meagre. Here, we asked whether the content of mental images, investigated by task complexity, is finely represented in the inhibitory interactions between the two primary motor cortices (M1s). Subjects’ left M1 was stimulated by means of transcranial magnetic stimulation (TMS) while they were performing actual or mental movements of increasing complexity with their right hand and exerting a maximum isometric force with their left thumb and index. Thus, we simultaneously assessed the corticospinal excitability in the right opponent pollicis muscle (OP) and the ipsilateral silent period (iSP) in the left OP during actual and mental movements. Corticospinal excitability in right OP increased during actual and mental movements, but task complexity-dependent changes were only observed during actual movements. Interhemispheric motor inhibition in the left OP was similarly modulated by task complexity in both mental and actual movements. Precisely, the duration and the area of the iSP increased with task complexity in both movement conditions. Our findings suggest that mental and actual movements share similar inhibitory neural circuits between the two homologous primary motor cortex areas.

## Introduction

Substantial experimental evidences argue for a functional equivalence between overt and covert states of voluntary actions. Notably, mental and actual movements engage similar neural structures [1]–[4], obey the same motor rules [5]–[7], and can improve, by means of pervasive repetition, motor performance [8]–[10]. Interestingly, transcranial magnetic stimulation (TMS) studies have shown that primary motor cortex (M1) is functionally relevant for mental movement simulation and motor learning by mental practice [11]–[18]. For instance, Debarnot and colleagues [18] have reported that virtual lesions of M1 dramatically prevent early gains in motor performance that are normally associated with motor imagery training.

Surprisingly, despite the significant involvement of M1 in mental actions, investigations on interhemispheric interactions between M1s are scarce [19], [20]. It is now well established that unimanual movement performance results in functional changes in both M1s [21]–[24] through transcallosal neural circuits [25]. Indeed, it has been demonstrated that during strictly unilateral hand movements the active M1, contralateral to the moving hand, exerts an inhibitory influence onto the ipsilateral M1, likely in order to suppress mirror activity [26]. The nature of interhemispheric interactions between M1s during mental actions remains to be elucidated. For instance, does a tennis player who mentally replicates an action trigger inhibitory processes between the two hemispheres similar to those during actual movements? To elucidate this question, we asked whether the content of mental images, here investigated by task complexity, is finely represented in the inhibitory interactions between the two M1s. Since task complexity finely modulates corticospinal excitability [27], [28] and is well integrated into the motor simulation process [29]–[31], one could expect also to influence interhemispheric interactions between M1s (see, [19], [20].

To this aim, we investigated the transcallosal inhibitory control exerted by the left to the right M1, measuring the iSP in the left hand while subjects performed actual or mental movements of increasing complexity with the right hand. Actually, interhemispheric motor inhibition can be evaluated by measuring the ipsilateral silent period (iSP), i.e., a brief interruption of voluntary EMG in a hand muscle by focal transcranial magnetic stimulation (TMS) of the ipsilateral M1 [32]–[34]. iSP is considered to be an original and particularly suited tool to investigate interhemispheric control of voluntary cortical motor output by measuring inhibition of volitional motor activity [19].

Based on the general idea that actual and mental states of actions trigger similar motor representations [3], we expected to observe a similar task-dependent modulation of iSP in both actual and mental actions.

## Material and Methods

### Ethical Statement

All participants gave their written informed consent prior to their inclusion in this study. The experimental protocol was approved by the ethics committee of Burgundy (AEC/B90097-40) and was carried out in agreement with legal requirements and international norms (Declaration of Helsinki, 1964).

### Participants and General Experimental Setup

Twelve healthy volunteers (seven males and five females; mean age = 27.8 yrs, range 23–38 yrs) participated in the experiments after given their written consent. All were good imagers, as they obtained scores higher than 43 (maximum score 56) in the French version of the Movement Imagery Questionnaire “MIQr” [35], and right handers, as individual scores were higher than 0.85 (Edinburgh Handedness Inventory [36]). In the current study, all measurements took place the afternoon (between 13∶00 h and 17∶00 h), because the temporal accuracy of mental movements reaches an optimum during this time of day [37]. Participants were isolated in a large room, which was temperature regulated (22±2°C) and illuminated with homogeneous white light. In all measurements, participants were comfortably seated on a chair with their head and arms fully supported.

### Interhemispheric Inhibition during Actual and Mental Actions of Different Complexity

**Figure 1:**
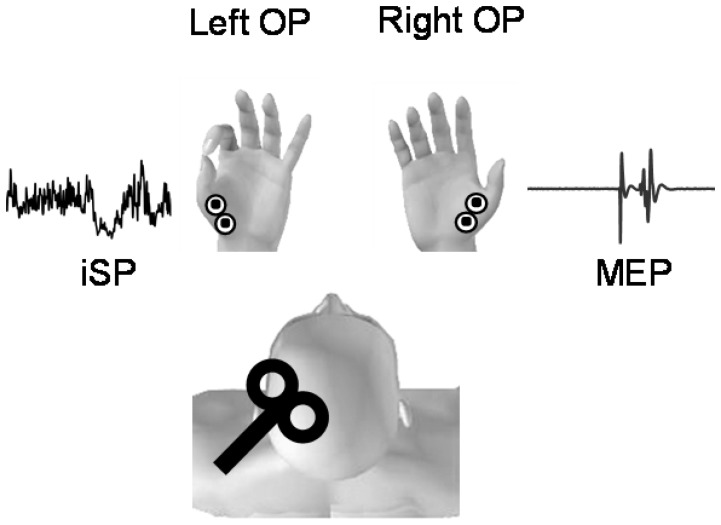
Experimental set up. Interhemispheric interactions are investigated through single pulse TMS over the right primary motor cortex. Participants exerted a maximum isometric contraction with the thumb and the index finger (i.e. thumb-index opposition) of their left hand, while they performed mental and actual movements with their right hand of increasing difficulty: full relaxation, opposition movements between thumb and index, and simple sequential movements between fingers.

We investigated to what extent mental or actual movements of different complexity involving the fingers of the right hand modulated the ipsilateral silent period (iSP) in the left Opponens Pollicis muscle (OP) (see Fig. 1). In our experimental design, participants exerted a maximum isometric contraction with the thumb and index finger (i.e. thumb-index opposition) of their left hand, while they were performing mental or actual movements with their right hand. Precisely, the right hand was involved in three tasks with increasing difficulty: full relaxation, mental and actual opposition movements between thumb and index, mental and actual sequential opposition movements between fingers.

- Full relaxation: participants were requested to totally relax their right hand. EMG activity from right OP was analyzed off-line (see below).
- Opposition movements between thumb and index: participants were requested to mentally and actually carry out repetitive right thumb-to-index opposition movements at 2 Hz (frequency was given by a metronome). The contact between the distal phalanxes of the thumb and index fingers corresponded to the beat sound.
- Sequential opposition movements between fingers: participants were requested to mentally and actually carry out repetitive right thumb-to-other fingers opposition movements at 2 Hz (metronome). The order of the finger movements in the sequence was: thumb-index- thumb-middle-thumb-ring-thumb-little.

Precise instructions were given to participants concerning mental movement simulation [38]. They were instructed to feel themselves (kinaesthesia) performing the motor task (first-person perspective) rather than watching themselves executing it (external imagery). Imagining a movement in the first person is a necessary condition to engage the motor system [39]. Specifically, they were asked to feel the motion of their fingers and the contact between the distal phalanx of the thumb and those of the other fingers following the tempo given by the metronome. After few practice trials, all participants declared being able to generate mental movements without difficulties.

Mental and actual movements were performed in a block design, separated by a 20 min time interval and counterbalanced between subjects. Within each session, motor tasks were randomized. In both sessions, the right and left arms were placed near to the trunk (shoulder abduction less than 5°), the forearms were supinated, the elbow angles were approximately 110° (full elbow extension: 180°), and the hands were aligned with the forearms. Each participant carried out 20 trials in each experimental condition. Each trial lasted between 4 s and 5 s, inter-trial intervals were at least 10 s and pauses were inserted whenever necessary.

### TMS Procedure

Single-pulses were delivered using a Magstim 200 stimulator (Magstim Co., Whitland, Wales, UK) with a monophasic current waveform connected to a figure-of-eight-shaped coil (external diameter of each loop, 9 cm) held tangentially to the scalp. The centre of the junction of the coil was placed over the hand area of the left M1 at the optimal position (hot spot) to elicit Motor Evoked Potentials (MEPs) in the contralateral OP, with the handle pointing backwards and 45° away from the midline. With this coil orientation, the induced current flowed in an anterior–medial direction approximately perpendicular to the central sulcus [40]. The corticospinal representation of OP was initially assessed with stimulator intensity regulated at 70% of its maximum power (2.2 T). The optimal coil location was searched by slightly moving the coil over the left M1 area until MEPs of maximal amplitude and lowest threshold in the right OP were elicited. The resting motor threshold (RMT) was defined as the intensity of stimulation needed to produce responses of approximately 50 µV in 50% of ten successive trials in the relaxed OP. Stimulation intensity was set at 120% of the RMT and in each experimental condition, 20 TMS pulses at random time-intervals were applied to the left M1 (i.e., one stimulation per trial). In each trial, the TMS was given ∼1 s after the beginning of the motor task. The pulse was timed-lock to the bit of the metronome that paced the motor task.

### EMG Recording

EMG activity was recorded from the right and left OP through pairs of surface electrodes glued to the participants’ skin according to a tendon-belly bipolar disposition. The EMG signals were amplified, filtered with a bandwidth ranging from 10 Hz to 1 kHz (using a 2nd order Butterworth filter), analogue-to-digital converted at a sampling frequency of 2 kHz and fed into a personal computer by means of the MP150 acquisition system (BIOPAC Systems Inc., Santa Barbara, CA, USA). Each recording epoch lasted 1.5 sec with 1 s preceded the single TMS pulse.

### Data Analysis

We analyzed the iSPs in the ipsilateral-left OP, the MEP in the contralateral-right OP, the average background EMG activity in the left OP during the 0.5 sec preceding the TMS pulse [19] and the RMS values in the right OP during the 0.1 sec preceding the TMS pulse.

Measurements of MEPs were made on single trials. The amplitude of contralateral MEPs (right OP muscle) was evaluated by taking the peak-to-peak difference in the raw EMG signals. However, the onset and the end of the iSP were difficult to define with precision in single trials. To overcome this difficulty, single trials (n = 20) were rectified and averaged within experimental condition and iSP duration was measured from the averaged trace [32], [33]. The onset of iSP was defined as the point after TMS at which EMG activity dropped constantly under the mean background EMG preceding the stimulus (i.e., the mean EMG calculated during the 0.5 sec before the TMS). The end of iSP was defined as the first point at which the level of EMG activity regained the mean EMG.

For each condition, the iSP duration was defined as follows:
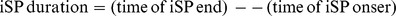



For each condition, the area of the iSP was calculated, using the following formula, where aur iSP is the area under rectified iSP:
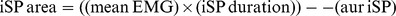



We calculated the RMS of EMG signals by using the following formula:


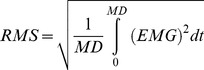


where MD is the movement duration.

### Statistical Analysis

Mean values of iSP duration, iSP area, MEPs amplitude, and EMG activity (RMS values) were calculated for each subject in each experimental condition. We tested that all variables were normally distributed (Shapiro-Wilk tests) and that sphericity was respected (Mauchly tests). We performed ANOVA with type of movement (actual and mental) and motor tasks (relaxation, opposition movements between thumb and index and sequential opposition movements between fingers) as within subject factors. Post hoc tests were performed using Tukey’s tests. Two-tailed paired t-tests were also performed whenever necessary. Statistical significance was accepted at P<0.05.

## Results

### Preliminary EMG Analysis

**Figure 2:**
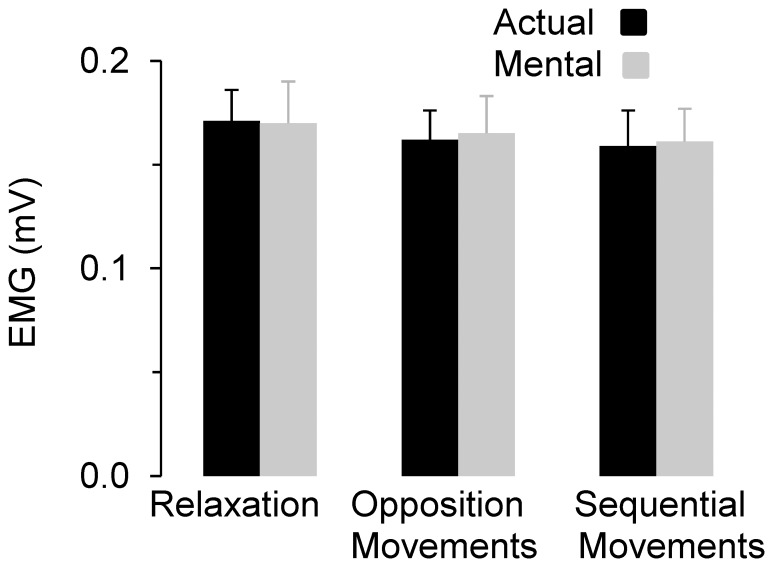
Average (± S.E.) background EMG level in the left OP for the different experimental conditions.

For the relaxation condition, we verified that participants did not activate their right OP muscle. Indeed, RMS analysis showed very low values (ranged from 0.003 mV to 0.013 mV) for all the participants. Further, we also evaluated that, during mental opposition movements between thumb and index and during mental sequential opposition movements between fingers, the right OP remained silent. Again, RMS analysis revealed very low values (ranged from 0.005 mV to 0.016 mV) for all the participants. Two-tailed paired *t-test* comparisons did not reveal significant differences between the full relaxation and the two tasks (in all cases, t<1.5, df = 11, P>0.4). In actual movements, we verified that EMG activity (RMS values) in the right OP during 0.1 sec preceding the TMS pulse was similar between the two tasks (opposition movements between thumb and index: 0.22±0.02 mV; sequential opposition movements between fingers: 0.21±0.02 mV; *t-tests*: t = 0.40, df = 11, P = 0.69). Furthermore, we also confirmed that EMG background activity in the left OP preceding the TMS pulse did not change according to the task. Indeed, average EMG level remained constant during the experimental conditions (see Fig. 2). ANOVA did not reveal any main (*type of movement*, P = 0.75; *motor task*, P = 0.21) or interaction effects (P = 0.97).

### iSP during Actual and Mental Actions of Different Complexity

**Figure 3:**
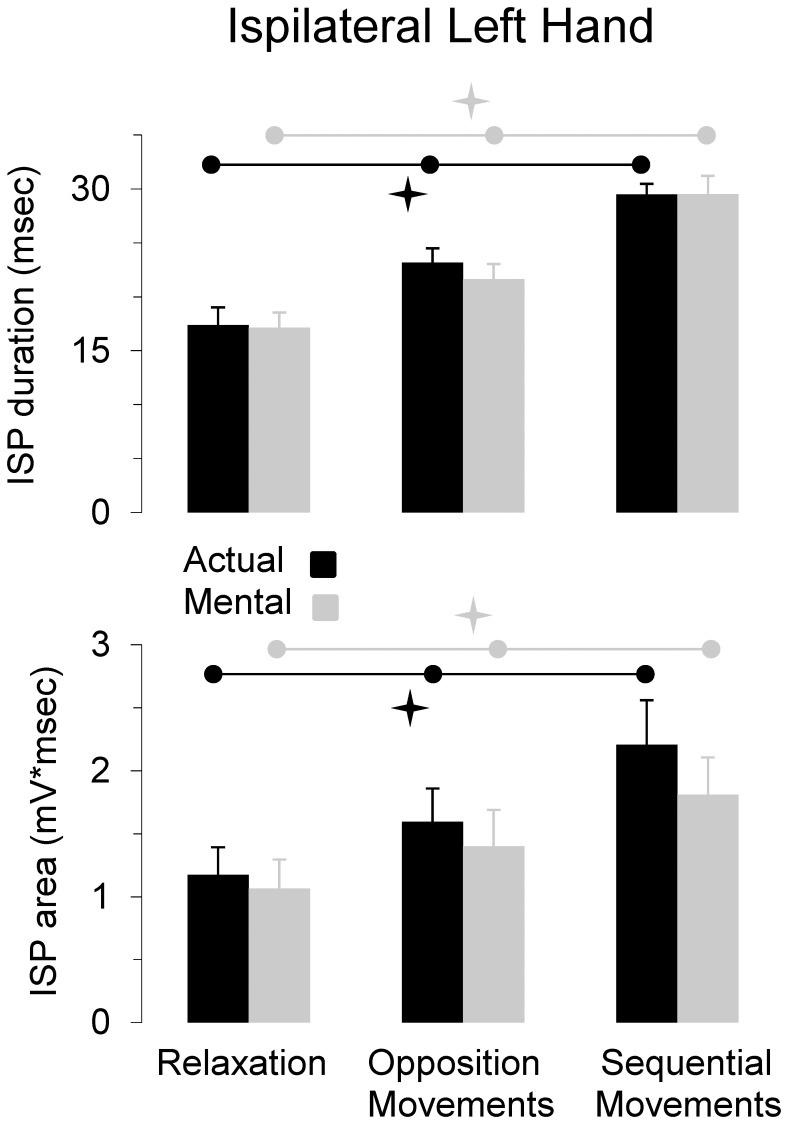
Average values (+SE) of iSP duration and iSP area recorded in the left OP for the mental and actual movements according to the motor tasks performed by the right hand. iSP duration and iSP area increase with task complexity. Horizontal black and grey bars and the corresponding stars indicate significant differences between the three conditions for both actual and mental movements.

**Figure 4:**
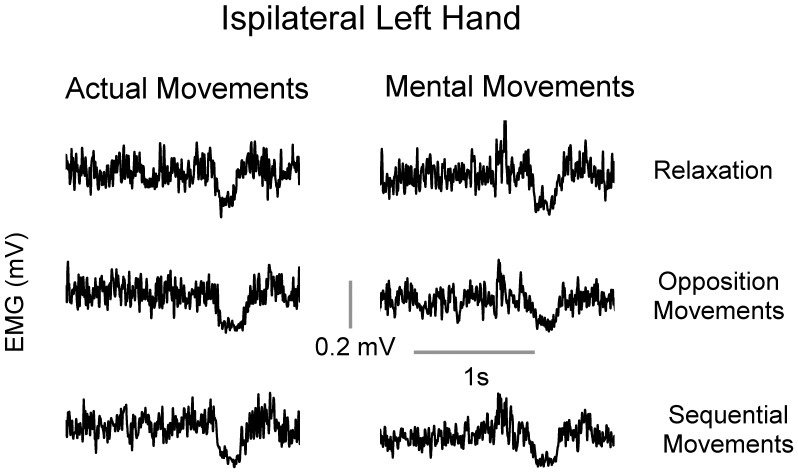
Typical EMG signals from the left OP muscle when the right hand is actually and mentally engaged in different motor tasks. EMG traces show iSP modulation according to task complexity.

Fig. 3 shows average values (+SE) for the iSP duration and iSP area for the three experimental conditions. For the iSP duration, ANOVA showed a main effect of *motor task* (F_2,22_ = 72.23, P<0.0001), but not a main effect of *type of movement* (F_1,11_ = 0.66, P = 0.43) or interaction effects (F_2,22_ = 0.41, P = 0.66). *Post hoc* analysis revealed that iSP duration increased according to motor task complexity in both actual and mental movements (in all cases, P<0.001). For the iSP area, we found similar results, namely a main effect of *motor task* (F_2,22_ = 32.31, P<0.0001), but not a main effect of *session* (F_1,11_ = 1.34, P = 0.27) or interaction effects (F_3,33_ = 1.26, P = 0.30). *Post hoc* analysis revealed that iSP area increased according to motor task complexity in both actual and mental movements (for all comparisons, P<0.05). Fig. 4 qualitatively illustrates the above findings by depicting typical EMG patterns from the left OP, in which the modulation of iSP with task complexity clearly appears for both actual and mental movements.

### MEP during Actual and Mental Actions of Different Complexity

**Figure 5:**
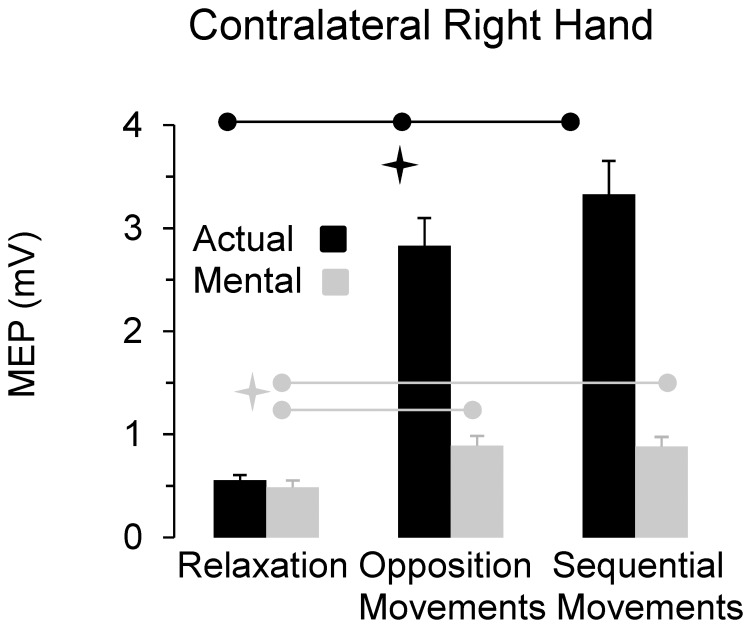
Average values (+SE) of MEP amplitudes from the contralateral right OP muscle. Horizontal black bars and the corresponding star indicate significant differences between the three conditions for the actual movements. Horizontal grey bars and the corresponding star indicate significant differences between the relaxation condition and the two others for the mental movements.

**Figure 6:**
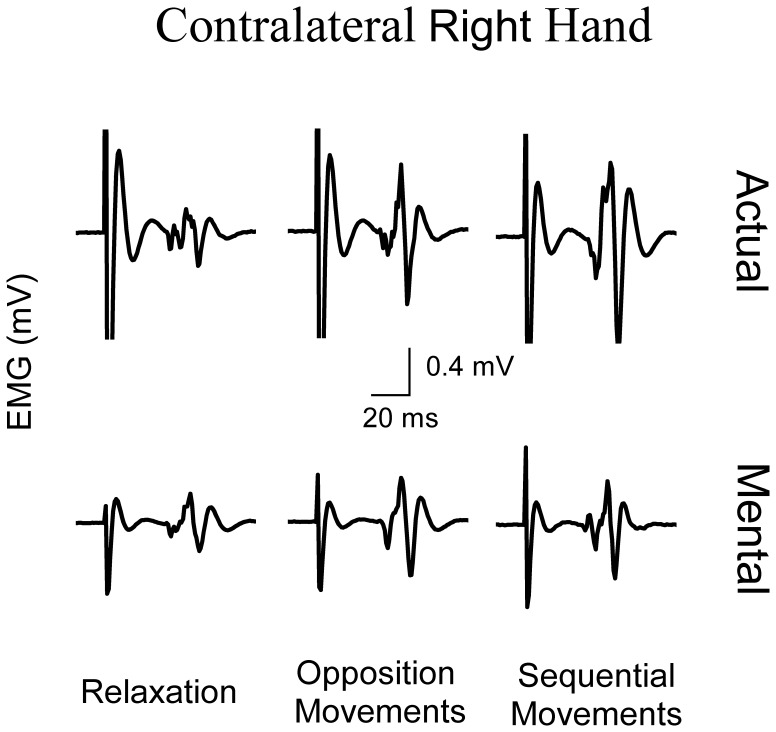
Typical MEPs from the right contralateral OP muscle when the right hand is actually and mentally engaged in different motor tasks.

Fig. 5 illustrates average values (+S.E) of peak-to-peak MEPs amplitude in the right OP for the different experimental conditions. ANOVA showed an interaction effect of *motor task* and *type of movement* (F_2,22_ = 43.65, P<0.0001). *Post hoc* comparisons showed that MEPs amplitude of both actual and mental movements significantly increased during the two motor tasks compared to the relaxation condition (in all cases, P<0.02). MEPs enhancement during mental actions testified that subjects were actively engaged in mental movement simulation. MEPs amplitude was greater for actual than mental movements in both motor tasks (in all cases, P<0.001), except for the baseline condition (P = 0.99). Interestingly, while MEPs amplitude increased with task complexity in actual movements (in all cases, P = 0.02), it remained constant in mental movements (P = 0.95). Fig. 6 qualitatively illustrates our finding regarding the contralateral right hand by depicting typical MEPs from the right OP.

## Discussion

In the current study, participants’ left M1 was stimulated by means of TMS, while they were performing actual or mental movements of increasing complexity with their right hand fingers and simultaneously were exerting a maximum isometric force with their left thumb and index. Corticospinal excitability in the contralateral right OP muscle increased during actual and mental movements, but task complexity-dependent changes were only observed during actual movements. Further, we found that interhemispheric inhibition in the ipsilateral left OP muscle was similarly modulated by task complexity in both mental and actual movements. Precisely, the duration and the area of the iSP increased with the increasing of task complexity in both movement conditions.

### iSP Modulation in the Ipsilateral Left Hand during Actual and Mental Actions of Different Complexity Involving the Contralateral Right Hand

Inhibitory interactions between the two M1s were comparable between mental and actual movements. We found that actual and mental finger movements of the right hand increased to the same extent the interhemispheric motor inhibition of the ipsiM1 measured as an enhancement of the iSP in the left OP muscle. Furthermore, we observed that task complexity significantly influenced the iSP in both movement conditions. Precisely, the higher was the complexity, the greater was the amount of inhibition evaluated by iSP. This forms an original finding and suggests that similar inhibitory circuits are involved in the control of the ipsiM1 during mental and actual movements. Although, mental actions involving finger movements have been already demonstrated to be able to enhance iSP with respect to rest condition [19], this is the first study showing that iSP could be modulated by task complexity in mental actions. Previous investigations have shown that mental images of different body parts activate the primary motor cortex in a somatotopic manner (Ehrsson et al. 2003). Our results go a step forward by showing that the content of mental images, here investigated by task complexity, is finely represented in the inhibitory interactions between the two M1s.

The increase in interhemispheric inhibition from the active to the opposite M1 during unilateral hand movements has been interpreted to play a role in suppressing mirror activity [19]. Our results could not be explained by changes in background EMG activity in the iSP target muscle (left OP), as the EMG level before the TMS pulse did not change across experimental conditions (cf. Fig. 2). Additionally, right OP was silent during mental actions and its EMG activity (RMS values before the TMS stimulation) was similar between the two tasks suggesting that iSP modulation according to task complexity cannot be attributed to a tiny muscle activation. Our findings expand the current knowledge on the interhemispheric inhibitory control of hand movements, because iSP measurements deal directly with interhemispheric inhibition of *voluntary* motor cortical output [19].

### MEPs Modulation in the Contralateral Right OP during Actual and Mental Actions of Different Complexity

We observed that corticospinal excitability of left M1 increased during actual and mental movements involving the right hand fingers. However, increase of excitability in left M1 was significantly lower during mental compared to actual finger movements as revealed by MEPs amplitude in the right OP. This finding corroborates those of previous studies [11]–[13], [41]–[43] and could be explained by the increase or absence of release of the inhibitory drive originating from many other cortical areas that prevent motor execution [18]. Indeed, interactions between the pre-motor areas, the posterior parietal lobe, and the primary motor cortex are facilitatory during overt movements and inhibitory during covert movements [44]. However, several studies demonstrated that the critical involvement of such parietal and premotor interactions in controlling the M1 excitability occur very early in the preparatory phases of movements [45]–[47], while the M1 excitability raises only from 100 ms before movement onset [48]. Hence we cannot exclude that the different facilitation of left M1 excitability observed during covert and overt movements may also be due to a fine-tuning of the activity of intracortical inhibitory circuits controlling the corticospinal pathway [15] [49]–[50].

In agreement with previous studies, we found that during actual unimanual movements corticospinal excitability of the contralateral active M1 changed with task complexity [27], [28], [51]–[53]. Indeed, corticospinal excitability in left M1 increased when subjects performed sequential movements involving the right fingers as compared to opposition movements between the right thumb and index. Note that RMS in the right OP during 0.1 s before the TMS stimulation was similar between the two tasks and therefore MEPs modulation according to task complexity cannot be attributed to an increase in the left M1 motor output. Conversely, in mental movements, the only significant change in left M1 corticospinal excitability was observed between the baseline (i.e., relaxation condition) and both the simple and complex tasks, but not within the motor tasks. This observation extends previous results, which showed no modulation of MEPs amplitude with mental simulation of graded isometric force [54], but is not completely in accordance with others showing significant differences in the corticospinal excitability between simple and complex imagined movements [55], [56]. This discordance could be due to different experimental paradigms used in these works. For example, in Roosink’s and Zijdewind’ study [47], participants had to learn a sequence of finger movements before mentally simulating it. Thus, the underlying neurophysiological processes are different from those involved during our protocol.

The lack of MEPs modulation with task complexity observed in our study could be explained by a ceiling effect due to the necessity of motor areas to keep the corticospinal excitability below a threshold over that intended movements become overt. We propose that subjects in the simplest task (i.e., opposition movements between thumb and index) may have already reached a value of M1 activity close to this threshold and that in the more complex task (i.e., sequential movements between fingers) a limited modulation of M1 activity was possible. In addition, if inhibitory interactions between pre-motor areas and M1 remain active during mental movements to prevent actual movements, the possible changes in the activation of M1 could be sometimes difficult to detect with neurophysiological and functional imaging studies [44].

### Functional Significance of Inhibitory Mechanisms during Mental Actions

It is now well admitted that internal forward models are engaged in motor imagery process [57], [58]. Forward models mimic the causal flow of the physical process by predicting the future sensorimotor state (e.g. position, velocity) given the efferent copy of the motor command and the current state of the motor system. Such a forward model scheme provides a parsimonious account of the tight temporal similarity between mental and actual movements [7], [29], [59]–[62]. To generate motor predictions during mental actions the forward internal model needs two sources of information: the initial state of the arm and the efferent copy of the motor commands. The former is provided by sensory signals from the periphery, while the latter by the activation of the motor cortex. There is increasing experimental evidence that the origin of the efference copy used by the brain to predict the future sensorimotor state is upstream from the motor cortex [63]. While actual and mental actions engage similar motor representations and sensorimotor predictions, they differ in their ultimate expression. In the first, selected motor plans are overtly executed; in the second, they must be prevented just before their actual implementation. Therefore, intracortical inhibition, observed in previous investigations [14]–[16], and interhemispheric inhibition, observed in the current and past studies [19], [20], is an important neural processing which helps brain to simulate movements and preserve intended actions from being overtly executed.

Here, a parallelism between mental actions and motor preparation could be done, although we consider that are not similar processes. Recent studies have proposed two concurrent inhibitory mechanisms during response preparation [64], [65]. The ‘impulse-control’ mechanism prevents selected responses from being emitted prematurely. The ‘competition-resolution’ mechanism helps to specify what response is required in a given context. We propose that mental actions could be controlled by similar inhibitory mechanisms. The ‘impulse-control’ mechanism could prevent overt activity in the hand involved in the mental action, while the ‘competition-resolution’ mechanism could suppress activity of the non-selected hand. These inhibitory mechanisms may allow the CNS to run the internal model and generate accurate predictions. The neurophysiological similarities between mental states and action preparation must be further elucidated. Is mental movement simulation the natural evolution of motor preparation?

### Chronobiological Considerations and Clinical Implications

As we have shown in previous studies, motor imagery process is modulated by circadian rhythms [37], [66]. In fact, a specific time window exists during the day (between 14 h and 20 h) where the duration of mentally simulated movements closely match the duration of executed movements. We have explained this *isochrony* between actual and mental movements in terms of a progressive calibration of internal models for action during the day, which ultimately leads to an optimum of the motor prediction process within the afternoon. Based upon such findings, it could be interesting to investigate the circadian modulation of interhemispheric excitatory and inhibitory processes during mental movement simulation.

Our findings could also be of interest for the field of motor rehabilitation. Based on the idea that motor imagery have a positive impact upon both motor performance [8]–[10] and functional reorganisation of the brain [67], [68], clinical studies have emphasized the efficiency of including mental movement tasks in specific rehabilitation protocols in order to improve motor function [69]–[72].
